# Fetal Microchimeric Cells in Blood of Women with an Autoimmune Thyroid Disease

**DOI:** 10.1371/journal.pone.0029646

**Published:** 2011-12-27

**Authors:** Trees Lepez, Mado Vandewoestyne, Shahid Hussain, Filip Van Nieuwerburgh, Kris Poppe, Brigitte Velkeniers, Jean-Marc Kaufman, Dieter Deforce

**Affiliations:** 1 Laboratory for Pharmaceutical Biotechnology, Ghent University, Ghent, Belgium; 2 Department of Endocrinology, University Hospital of Brussels (VUB), Brussels, Belgium; 3 Department of Endocrinology, Ghent University Hospital, Ghent, Belgium; Cardiff University, United Kingdom

## Abstract

**Context:**

Hashimoto's thyroiditis (HT) and Graves' disease (GD), two autoimmune thyroid diseases (AITD), occur more frequently in women than in men and show an increased incidence in the years following parturition. Persisting fetal cells could play a role in the development of these diseases.

**Objective:**

Aim of this study was to detect and characterize fetal cells in blood of postpartum women with and without an AITD.

**Participants:**

Eleven patients with an AITD and ten healthy volunteers, all given birth to a son maximum 5 years before analysis, and three women who never had been pregnant, were included. None of them had any other disease of the thyroid which could interfere with the results obtained.

**Methods:**

Fluorescence *in situ* hybridization (FISH) and repeated FISH were used to count the number of male fetal cells. Furthermore, the fetal cells were further characterized.

**Results:**

In patients with HT, 7 to 11 fetal cells per 1.000.000 maternal cells were detected, compared to 14 to 29 fetal cells in patients with GD (*p* = 0,0061). In patients with HT, mainly fetal CD8^+^ T cells were found, while in patients with GD, fetal B and CD4^+^ T cells were detected. In healthy volunteers with son, 0 to 5 fetal cells were observed, which was significantly less than the number observed in patients (*p*<0,05). In women who never had been pregnant, no male cells were detected.

**Conclusion:**

This study shows a clear association between fetal microchimeric cells and autoimmune thyroid diseases.

## Introduction

Autoimmune diseases affect approximately 5–8% of the population and of all the subjects with an autoimmune disease, 78% are women [1]. Many hypotheses have been proposed to explain this gender bias: differences in cytokine and hormone production in men and women, and/or differences in the degree of immune response which tend to be more vigorous in females, resulting in a higher antibody production and cell-mediated immunity after immunization [2].

Another explanation might be found in the postpartum presence of fetal cells in the maternal circulation and tissues. During pregnancy, fetal cells cross the placenta into the maternal circulation [3], [4]. The immunological interaction between maternal and fetal immune cells should at that point be minimal or negligible [5]. Fetal cells can persist in the postpartum period, which indicates insufficient elimination after delivery[6]. These cells reside in maternal blood and tissues such as the skin and the thyroid [7], [8], [9], [10], [11]: the mother becomes microchimeric. The persistence of these fetal cells may result in the development of autoimmune diseases that affect women postpartum, such as autoimmune thyroid diseases (AITD) [12], [13]. This assumption is based on the higher incidence of these diseases in women in the decades that follow parturition and on their similarities with graft-versus-host disease after haematopoietic cell transplantation, an iatrogenic form of chimerism [13], [14].

Autoimmune thyroiditis and Graves' disease are two autoimmune thyroid diseases, affecting 5–15% of women. In patients with autoimmune thyroiditis, specific auto-antibodies in serum are present, including anti-thyroid peroxidase antibodies (TPOAb), anti-thyroglobulin antibodies (TgAb) and autoantibodies binding to the TSH receptor (TSHRAb). Patients with hypothyroidism and goiter have Hashimoto's thyroiditis (HT). A variant of HT is atrophic thyroiditis. These patients present with hypothyroidism and atrophic thyroid [15]. Patients with HT can also present with euthyroidism. Graves' disease (GD) is characterized by the presence of circulating autoantibodies that bind and activate the thyrotropine receptor (TSHRAb), stimulating follicular hypertrophy and increases in thyroid hormone production resulting in hyperthyroidism [16], [17]. These patients can also have TgAb and/or TPOAb [18].

HT and GD are more prevalent in women between the ages of 30 and 50 years, with a ratio female:male of respectively 10∶1 and 7∶1, and are often detected in the years following parturition [17], [19], [20]. Therefore, our study focused on these two autoimmune thyroid diseases.

It has been hypothesized that within the thyroid, the presence of fetal cells may initiate an immune response resulting in an AITD [5], [19]. However, direct evidence for such an effect is lacking. To our knowledge, no studies so far have described the presence of fetal microchimeric cells in the maternal circulation of patients with an AITD in the decades that follow parturition. Moreover, no studies have examined which cell types represent the fetal cell fraction in the blood of these patients. The aim of this study was to compare the amount of fetal cells in peripheral blood of women with and without an autoimmune thyroid disease, in the years that follow parturition. Furthermore, the detected fetal cells were characterized.

## Methods

### Ethics Statement

This study was approved by the Ethics Committee of the Ghent University (B67020095877), Belgium, and written informed consent was obtained from all participants.

### Study participants

The diagnosis of Hashimoto's thyroiditis was based on the presence of thyroid antibodies (TgAb, TPOAb) and hypothyroidism. The diagnosis of Graves' disease was based on the presence of hyperthyroidism, eventually diffuse goiter, and positive serum TSH receptor antibodies. Peripheral blood (PB) was collected from 11 patients with an AITD, who had given birth to a son maximum five years before analysis. Blood was also obtained from 10 healthy volunteers who had given birth to a son maximum five years before analysis and from 3 women who never had been pregnant nor had a transfusion or transplantation. In addition, PB was collected from two women who were pregnant during the course of the study. Peripheral blood was taken just before birth of their son, 1 week and 6 months postpartum. Extreme precautions were taken to avoid external contamination. In particular, all samples were handled by a female laboratory technician.

### Fluorescence in situ hybridization (FISH)

Peripheral blood mononuclear cells (PBMCs) were isolated from the patient's EDTA blood samples by density gradient centrifugation on Ficoll-Paque Plus (GE Healthcare, Diegem, Belgium) according to the manufacturer's instructions. From each sample, 1.000.000 PBMCs were cytospun on 4 glass slides as previously described[21]. The slides were air dried and fixated for 5 minutes in a Carnoy's fixative (3∶1 methanol (Fisher scientific, Leicestershire, UK): acetic acid (Sigma-Aldrich, Bornem, Belgium)).

**Figure 1:**
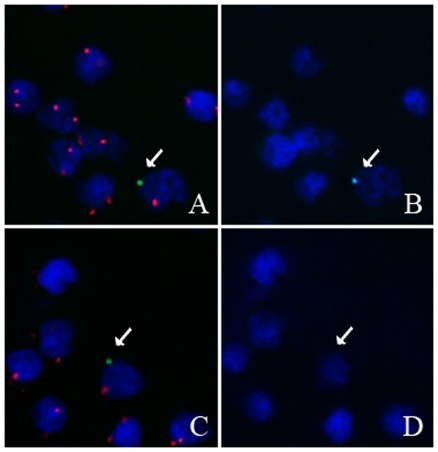
FISH and Repeated FISH. A. FISH of female cells, showing two SpectrumOrange X FISH spots and of a presumed male cell indicated by an arrow, showing one SpectrumOrange X FISH and one SpectrumGreen Y FISH spot; B. Repeated FISH of the female cells and the presumed male cell, showing no SpectrumAqua Y FISH spots in the female cells. In contrary, the male cell shows one SpectrumAqua Y FISH signal on the exact same location as the SpectrumGreen Y FISH spot in image A (indicated by an arrow), indicating this is a true male cell. C. FISH of female cells and of one presumed male cell, indicated by an arrow. D. Repeated FISH of a male cell (C) shows no SpectrumAqua Y FISH spot. The SpectrumGreen Y FISH spot was probably caused by cellular debris or dust particles. The SpectrumOrange X FISH spot of that cell is larger than the other SpectrumOrange spots which may indicate two SpectrumOrange X FISH spots lying very closely to each other.

Male fetal cells were distinguished from maternal cells by fluorescence *in situ* hybridization (FISH) with CEP X SpectrumOrange/CEP Y SpectrumGreen DNA probes (Vysis, Abbott Molecular, Illinois, US). Male cells showed one SpectrumGreen Y FISH dot and one SpectrumOrange X-FISH dot, while female cells contained two SpectrumOrange X FISH dots (Figure 1). FISH was performed following the manufacturer's instructions with minor adjustments. Samples were incubated in a 0,01% pepsin (Serva Electrophoresis, Heidelberg, Germany)/0,01M HCl (Sigma-Aldrich)-solution during 30 minutes at 37°C and washed with PBS (Invitrogen, Paisley, UK) and washing buffer (1x PBS, 0,5M MgCl_2_ (Sigma-Aldrich)). In the next step, cells were fixated for 10 min in 1% formaldehyde (Acros Organics, Geel, Belgium), rinsed with PBS, dehydrated for 3 minutes using an ethanol series (70%, 90% and 99%, Merck, Darmstadt, Germany) and air-dried. Afterwards, DNA was denatured by heating the slides in a denaturation solution (70% formamide (Sigma-Aldrich), 2x SSC (Vysis)) for 5 minutes at 73°C. Slides were dehydrated again for 1 min using an ethanol series (70%, 90% and 99%). Slides were dried on a hot plate (50°C) and 5 µl of the pre-denatured probe-mixture was added per slide. After applying a coverslip, hybridization was performed at 42°C overnight. Subsequently, slides were rinsed for 5 min with preheated 0,4x SSC/0,1% NP-40 (Sigma-Aldrich) solution at 73°C and three times for 2 minutes at room temperature (RT) with 2x SSC/0,1% NP-40. After air-drying, the slides were mounted with antifade Vectashield mounting solution (Vector Labs, Burlingame, CA, USA) containing 4′,6-diamidino-2-phenylindole dihydrochloride (DAPI, 400 ng/ml, Sigma-Aldrich) to counterstain all nuclei on the slide. A coverslip was applied.

### Fluorescence scanning

The scanning stage was controlled by the AxioVision 4.6.3 software (Carl Zeiss, München, Germany), using the MosaiX module. Image acquisition was carried out with the AxioVision multichannel fluorescence module and the AxioCam MRm camera (Carl Zeiss). Cell nuclei were visualized using Zeiss filter set no. 49 (G 365 nm, FT 495, BP 445/50), Y chromosome spots with Zeiss filter set no. 38 (BP 470/40, FT 495, BP 525/50) and X chromosome spots with filter set no. 20 (BP 546/12, FT 560, BP 575–640). Slides were scanned at 20x magnification using a Carl Zeiss short distance Plan-Apochromat® objective [21]. From every slide, 582 images were acquired and were stored as separate tiff-files.

### Segmentation and masking

**Figure 2:**
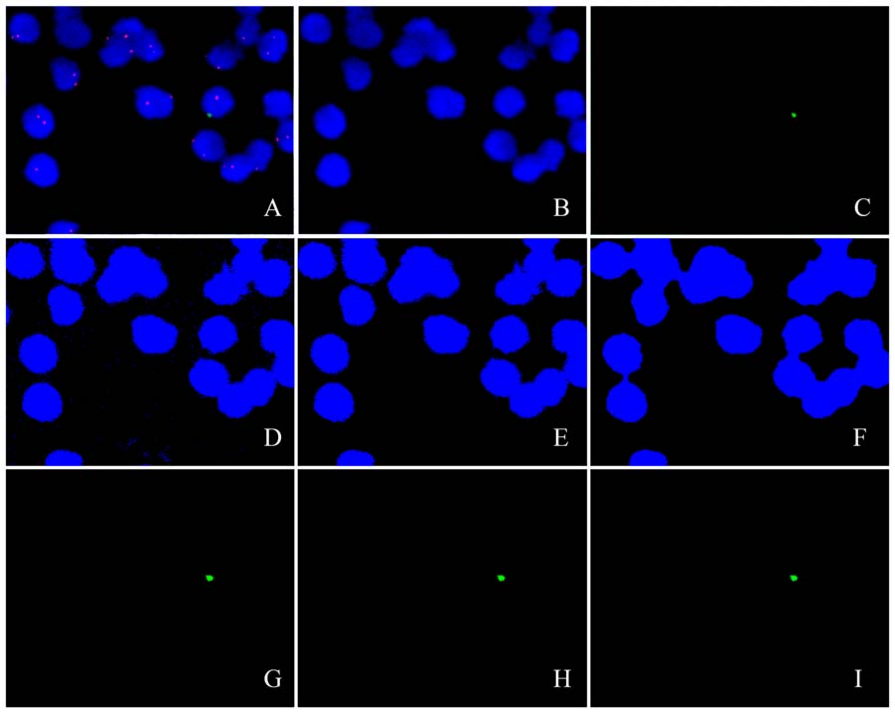
AxioVision Commander script for the automatic detection of male fetal cells. A. Original image, split up in B. DAPI image and C. SpectrumGreen image; D. Threshold interactive on the DAPI image: segmentation based on the definition of a brightness range. Pixels within the defined gray level range are set to the maximum gray value 1 (pseudocolor blue); whilst pixels outside it are set to the minimum gray value 0 (black), resulting in a binary image; E. Scrap of image D: removing all artefacts too small to be possibly originating from cell nuclei; F. Close: filling in gaps in the contours of the nuclei; G. Dynamic Threshold of the SpectrumGreen image, resulting in a binary image showing the Y chromosome FISH spots; H. Scrap of image G: all regions smaller than 10 pixels and larger than 65 pixels are removed; I. Masking of the binary images F and H: retaining only the SpectrumGreen FISH signals lying in a nucleus. In the last step, to be included as a true FISH signal, the detected regions had to fulfil four measurement parameter conditions with regard to area of the region, lowest and highest pixel density and standard deviation of the pixel density (not shown).

For automatic detection of the male fetal cells, the image processing AxioVision Commander module (Carl Zeiss) was used. All steps of processing, analysis, and evaluation were stored in an AxioVision Commander Script, which could be run automatically on the stored images. This script was based on previously published scripts with some specific modifications (Figure 2)[21], [22]. SpectrumOrange X chromosome FISH signals were used as a visual control. The validation of the script has been described earlier [21].

### Repeated FISH

Results of FISH were confirmed using another CEP Y FISH probe labelled with SpectrumAqua (Vysis). The repeated FISH protocol was performed as described by Liu *et al.* with a few minor modifications [23]. Cover slips applied after FISH were washed off in water. Slides were incubated for 10 min in each solution of 60% formamide/2x SSC solution; 2x SSC and 4x SSC/0,1% NP-40 solution at 50°C. Slides were dehydrated for 1 min through an ethanol series (70%, 90% and 99%) at RT and air-dried. Denaturation, hybridization and subsequent washing steps were performed as described above. Results of the repeated FISH were visualised by using the Zeiss filter set no. 47 (BP 436/20, FT 455, BP 480/40) (Figure 1 B).

### Phenotyping of the fetal microchimeric cells

For 6 patients with an AITD, phenotyping of the fetal microchimeric cells was performed. After isolation of the PBMCs, B, CD4^+^ T and CD8^+^ T cells were enriched using an EasySep positive selection strategy (Stemcell Technologies, Vancouver, Canada) according to manufacturer's instructions. Purity of the different fractions was determined with flow cytometry. Cells were stained with monoclonal antibodies against CD3 (labelled with PE-Cy5; 1∶20), CD4 (FITC; 1∶20), CD8 (PE-Cy7; 1∶20) and CD19 (PE; 1∶20) (eBiosciences) for 30 minutes on ice, in the dark [24]. All analyses were performed on a Cytomics FC500 flow cytometer (Beckman Coulter, Miami, Florida, US) and data analysis was performed by CXP analysis software (Beckman Coulter). These cell fractions were also cytospun on poly-L-lysine slides and underwent FISH and repeated FISH as described above.

### Statistical analysis

Levels of significance were calculated by SPSS (IBM, New York, US) using Mann Withney (MW) test. *p*<0,05 was regarded as significant.

## Results

### Study participants

A medical history concerning former pregnancies, transplantations and blood transfusions which can influence the results of microchimerism was available for patients (Table 1) and healthy volunteers (Table 2). Patients with an AITD and healthy volunteers, both with a son, were of similar age (mean 32,1 yr (range 25–37) for patients and mean 31,1 yr (range 26–39) for healthy volunteers; *p* = 0,41). Three healthy volunteers who never were pregnant and two healthy volunteers who were pregnant at the conduct of the study, were younger (mean 26,4 yr (range 25–27 yr); *p* = 0,015). Patients and healthy volunteers with son, had similar numbers of children (mean 1,4 (1–2) versus 1,5 (1–2); *p* = 0,69) and similar number of boys (mean 1 versus 1,2 (1–2)); which were of similar ages (mean 32,4 months (10–68 months) versus 36,3 months (3–91 months); *p* = 0,85). None of the patients and healthy volunteers had any other disease of the thyroid. There was no significant difference in the amount of isolated PBMCs/ml PB between patients and healthy volunteers (data not shown).

**Table 1:** Patients' information possibly relevant to fetal microchimerism.

Patient	AITD	Age patient (years)	Diagnosis since birth of youngest son (months)	Age (months)* and sexes of the children	Miscarriage	Transfusion	Additional information considering the thyroid	Fetal cells/ 1.000.000 maternal cells
**1**	HT	32	10	10; male	-	-	-	11
**3**	HT	35	12	39; male, 16; female	3× very earlyin pregnancy	-	Aunt with thyroid dysfunctions	8
**5**	HT	25	8	16; male	-	-	-	8
**6**	HT	35	3	51; male, 87; female	5 yrs ago	-	-	7
**7**	HT	36	4	26; male	-	-	Mother with hypothyroid	7
**9**	HT	29	unknown	15; male	-	-	-	9
**11**	HT	32	unknown	18; male	2 x, 1 and 3 years ago	-	Mother and grandmother with hypothyroid	10
**2**	GD	37	12	37; male	6,5 months old death born son 4 yrs ago	-	Aunt with hyperthyroid	29
**4**	GD	32	4	25; male	-	9 yrs ago	-	15
**8**	GD	32	unknown	68; male, 8; female	-	-	-	14
**10**	GD	28	5	37; male, 123; female	-	-	-	21

*within the conduct of the study.

**Table 2:** Healthy volunteers' information possibly relevant to fetal microchimerism.

Healthy Volunteer	Age volunteer (years)	Age (months)* and sexes of the children	Miscarriage	Transfusion	Additional information considering the thyroid	Fetal cells/1.000.000 maternal cells
**1**	31	43; male	1.5 yr ago at 7 weeks	-	-	3
**2**	39	91; male, 59; male	-	-	-	3
**3**	34	45; male, 20; male	-	-	Sister with possibly GD	3
**4**	26	6; male	-	-	-	1
**5**	29	54; male	-	-	-	1
**6**	29	33; male, 7; female	17m ago	-	-	1
**7**	32	32; male, 68; female	-	-	-	5
**8**	31	50; male, 89; female	-	-	-	1
**9**	33	3; male	2x; 2 and 1 yr ago	-	-	0
**10**	27	6; male	-	-	-	1
**Pregnant 1, 1 week before delivery**	27	-	-	-	-	2
**Pregnant 1, 1 week postpartum**	27	0; male	-	-	-	2
**Pregnant 1, 6 months postpartum**	28	6; male	-	-	-	1
**Pregnant 2, 1 week before delivery**	26	-	-	-	-	1
**Pregnant 2, 1 week postpartum**	26	0; male	-	-	-	1
**Pregnant 2, 6 months postpartum**	26	6; male	-	-	-	1
**Negative control 1, never pregnant**	26	-	-	-	-	0
**Negative control 2, never pregnant**	25	-	-	-	-	0
**Negative control 3, never pregnant**	27	-	-	-	-	0

*within the conduct of the study.

### Fetal microchimerism in patients and healthy volunteers

**Figure 3:**
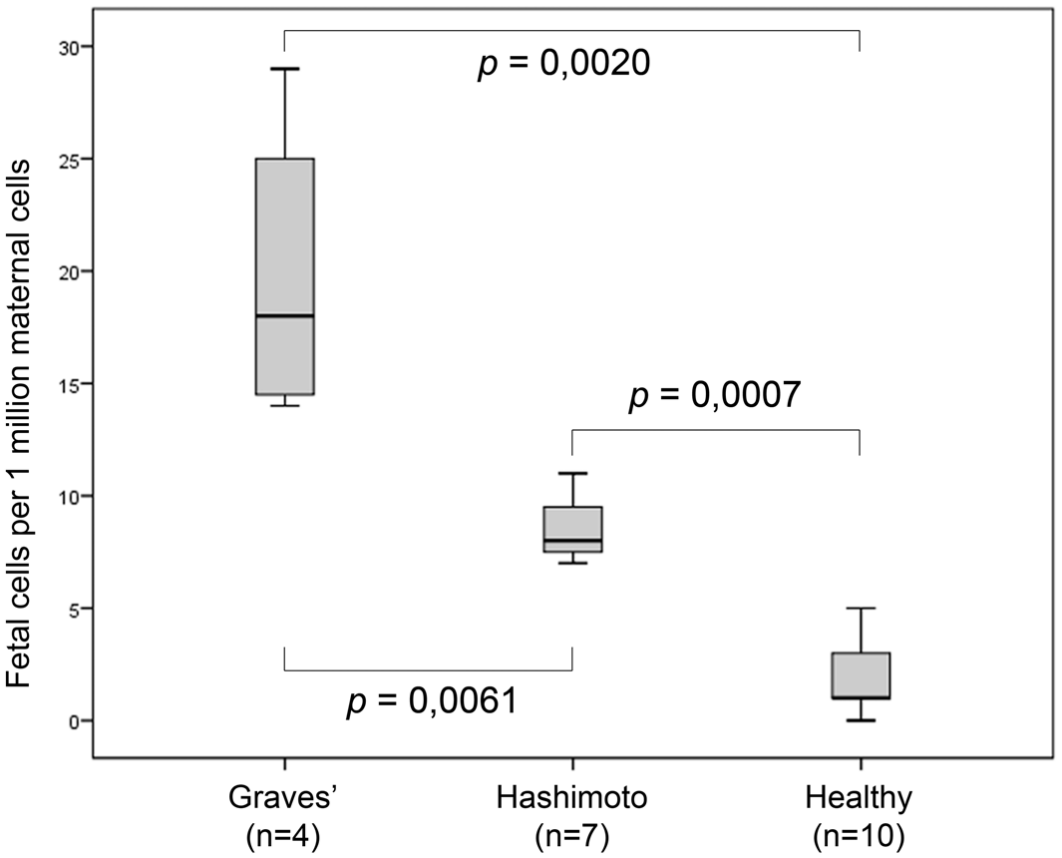
Boxplot: fetal cells in patients with GD or HT, and healthy volunteers with son. Minimum and maximum numbers of detected fetal cells are shown, as well as first quartile, median and third quartile. The number of fetal cells was significantly different between the three groups (*p* <0,05). Moreover, a significant difference between patients with GD and patients with HT was observed (MW, *p* = 0,0061).

The number of fetal cells detected in patients with HT or GD and healthy volunteers is shown in Table 1 and 2 respectively. All patients had detectable fetal microchimerism in their PB, ranging from 14 to 29 fetal cells per million maternal cells for patients with GD and from 7 to 11 fetal cells per million maternal cells for patients with HT. In all healthy volunteers who gave birth to a son, except for one, fetal microchimeric cells were found in PB and ranged from 1 to 5 fetal cells per million maternal cells. There was a statistically significant difference between patients with GD and patients with HT compared to healthy controls (respectively *p* = 0,002 and *p* = 0,0007, MW) (Figure 3). Moreover, patients with GD had significant more fetal cells in their blood compared to patients with HT (MW, *p* = 0,0061).

Blood obtained from two women pregnant of a boy, revealed respectively 2 and 1 fetal cell(s) per million maternal cells. One week postpartum, these women had the same amount of fetal cells in their blood as before delivery (2 respectively 1). Blood was also obtained 6 months postpartum and revealed in both women 1 fetal cell per million maternal cells (Table 2).

As every woman had given birth to a son, the origin of the male cells is likely to be fetal. However, persistent microchimerism occurring after blood transfusion or transplantation has also been described [25], and this possibility cannot be ruled out for patient 4 (GD) who had a blood transfusion 9 years ago. However, as the results of this patient did not differ from the results of other patients with Graves' disease, and hence inclusion or exclusion didn't influence the results, this patient was included in our study but results for this specific patient should be interpreted carefully.

The negative control group, consisting of three women who had never been pregnant nor had a transfusion or abortion, was consistently negative for male cells (Table 2).

### Flow cytometry for determination of purity of EasySep isolated cells

Purity of the EasySep isolated cells was assessed by flow cytometry (Beckman Coulter Cytomic FC 500). For the isolation of B cells, CD4^+^ T cells and CD8^+^ T cells, a purity of respectively 97,6%, 97,4% and 94,1% was obtained (data not shown).

### Fetal microchimeric cells in PBMC subsets

All patients selected for our analysis were positive for male cells in unsorted PBMCs. Table 3 shows the distribution of the fetal cells in the different cell subtypes (CD4^+^ T, CD8^+^ T, B cells and other cell types) for 6 patients with an AITD (patient 2, 7, 8, 9, 10 and 11). For patient 5, only fetal T cells were detected (9,3 fetal cells per 1 million maternal T cells) and no subdivision into CD4^+^ and CD8^+^ T cells could be made, due to sample amount limitation. No fetal B cells or other fetal cell types were detected in this patient (data not shown in table 3).

**Table 3:** Fetal cells in the sorted cell fractions in patients with HT (patient 7, 9 and 11) and GD (patient 2, 8 and 10).

Cell type		Pat 7 (HT)	Pat 9 (HT)	Pat 11 (HT)	Pat 2 (GD)	Pat 8 (GD)	Pat 10 (GD)
**B cell**	CD19^+^	0	3,4	1,2	4,0	4,0	9,0
**T cell**	CD4^+^	0	1,5	1	1,0	2,5	9,7
	CD8^+^	4,7	6,0	3,4	1,5	0,5	9,3
**Other cell types**		0	0	0	0	0	9,3

Data are represented as normalized counts of fetal cells per 1 million maternal cells. T cells were split up immediately into CD4+ and CD8+ T cells. B and T cells were positively isolated with the EasySep® isolation kits. Cells not isolated with T or B cells, formed the cell population ‘other cell types’. B cells were counted in a range from 1.000.000 to 2.000.000 cells, CD4^+^ T cells ranged from 1.000.000 to 2.250.000 and CD8^+^ T cells from 1.500.000 to 2.000.000.

In patients with GD, the majority of the fetal cells were found in the B cell fraction and CD4^+^ T cell fraction while in patients with HT, fetal cells were mainly CD8^+^ cytotoxic T cells.

## Discussion

Hashimoto's thyroiditis and Graves' disease, two autoimmune thyroid diseases, occur more often in women than in men and show an increased incidence in the decades that follow parturition [10], [17], [19], [20]. It has been hypothesized that fetal microchimeric cells play a role in the development of these diseases [5], [26], [27]. Although fetal microchimerism has already been shown in the thyroids in 50% of these patients, no studies to date have detected and characterized fetal cells in peripheral blood, although blood from these patients is easier to obtain in contrast to thyroid tissue [9], [11], [26], [27]. Studies describing the presence of fetal cells in thyroid glands, often do not mention the reason of removal of the thyroid gland [9], [27]. Srivatsa *et al.* analysed thyroid glands from patients with HT removed because of follicular neoplasm or papillary carcinoma [11]. The presence of tumor cells may confound the analysis and the results [9], [27], [28]. Therefore, only patients without any other disease of the thyroid were included in our study. Our study focused only on women who had given birth to a son since male fetal cells are easier to detect in contrast to female fetal cells.

To examine the presence of fetal cells in autoimmune thyroid diseases, blood from female patients with GD or HT who had given birth to a son maximum five years before analysis, was assessed in this study. Using FISH and repeated FISH as an additional confirmation, the number of fetal cells in patients was compared with that detected in healthy volunteers. All patients with an autoimmune thyroid disease included in our study had detectable fetal microchimerism in their blood. This is contrary to results obtained in the thyroid glands where only 50% of the patients had fetal microchimerism. The highest number of fetal cells was observed in the unsorted PBMC fraction of patients with GD (14 to 29 fetal cells per million maternal cells), followed by HT (7 to 11) compared to the low number of fetal cells detected in healthy volunteers (0 to 5). This indicates a higher degree of microchimerism in AITD compared to healthy controls. Moreover, significant more fetal cells were detected in patients with GD compared to patients with HT (*p* = 0,0061).

Two additional control groups were included in our study. Analysis of the blood of two pregnant women revealed respectively 1 and 2 fetal cells per million maternal cells. This corresponds to the number of fetal cells detected in our healthy group and the amount detected by Bianchi *et al.* [6]. As a negative control group for this study, blood from women who never were pregnant nor had a transfusion or transplantation, was obtained. No male cells were detected in their blood, which gives strong evidence of the reliability of the techniques used in our study.

As a significant difference in fetal cells was found between patients with HT and GD, it can be presumed that fetal cells have a different role in the pathogenesis of both diseases. Our study focused on the presence of fetal B and T cells because these subsets are more likely to initiate or be involved in immune response. In patients with HT, mainly fetal CD8^+^ cytotoxic T cells were found. In patient 5, only fetal T cells were detected (9,3 fetal cells per million maternal cells). In patient 7, fetal cells were only detected in the CD8^+^ T cell fraction (4,7 fetal cells per million maternal cells). In patient 9 and 11, the majority of fetal cells was composed of CD8^+^ T cells (respectively 6 and 3,4 fetal cells per million maternal cells). The remaining fetal cells for these patients consisted of CD4^+^ T cells (respectively 1,5 and 1 fetal cell(s) per million maternal cells) and B cells (respectively 3,4 and 1,2 fetal cells per million maternal cells). One might speculate that these cytotoxic T cells could cause cell death leading to hypothyroidism[29]. In GD however, the majority of fetal cells was found in the B cell fraction (4 fetal cells per million maternal cells for patient 2 and patient 8). These B cells could possibly be activated by fetal CD4^+^ T cells (1 fetal cell per million maternal cells and 2,5 fetal cells per million maternal cells respectively for patient 2 and 8). In one patient with GD (patient 10), more fetal T cells (9,7 fetal CD4^+^ T cells per million maternal cells and 9,3 fetal CD8^+^ T cells per million maternal cells) than fetal B cells (9 fetal cells per million maternal cells) were found, along with some other cell types (9,3 fetal cells per million maternal cells). These other cell types were cells not isolated during selection of the T and B cells and are likely to be natural killer (NK) cells or hematopoietic progenitor cells capable of differentiating into immune competent cells[30]. One might speculate that thyroid-reactive T cells could cause activation of thyrotropin receptor (TSHR)-reactive B cells, secreting TSHR-stimulating antibodies causing hyperthyroidism [29]. These thyroid antibodies have already been described in blood [31].

Fetal microchimeric cells could play a role in the pathogenesis of AITD in two ways: direct or indirect. In a direct manner, fetal lymphoid cells migrating into the thyroid could initiate a graft versus host reaction against maternal thyroid antigens resulting in an autoimmune thyroid disease [5], [13]. On the other hand, intrathyroidal fetal cells, not necessarily of the lymphoid lineage, could indirectly be involved in the pathogenesis of autoimmune thyroid disease by activating intrathyroidal maternal T cells against fetal antigens. Despite the fact that the fetal cells were only characterized in a limited number of patient samples, an increase in fetal lymphocytes was clearly shown, which provides support for the first hypothesis.

In conclusion, our findings indicate a significant difference in number of fetal cells in the maternal circulation of patients with an AITD and healthy volunteers despite similar age, number and gender of their children. In addition, a significant difference was found between patients with GD and HT, where patients with GD had more fetal cells in their blood circulation. Moreover, the fetal cells were of a different cell type which might possibly correlate to the pathogenesis of both diseases. Our study shows a clear association between fetal microchimeric cells and autoimmune thyroid diseases and indicate the value and need for further research in this field.
